# Thyrotoxicosis and Moyamoya Syndrome: A Fatal Intersection of Rare Disorders in a Young Patient With a Background of Hypothyroidism

**DOI:** 10.7759/cureus.100852

**Published:** 2026-01-05

**Authors:** Hussein Abu Rabia

**Affiliations:** 1 Medicine and Surgery, Chelsea and Westminster Hospital NHS Foundation Trust, London, GBR; 2 General Medicine, Manchester Foundation NHS Trust, Manchester, GBR

**Keywords:** euglycemic diabetic ketoacidosis, hypothyroid disorders, moyamoya disease and syndrome, risk of seizure, severe thyrotoxicosis, stroke, thyroid storm

## Abstract

Thyroid storm, also known as thyrotoxic crisis, is an uncommon and potentially life-threatening condition that can cause metabolic, cardiovascular and neurological complications. Thyrotoxic crisis often develops in the context of untreated or poorly controlled hyperthyroidism and is typically precipitated by acute stressors such as infection, surgery, or trauma. While the progression from hypothyroidism to hyperthyroidism is itself an uncommon clinical occurrence, the emergence of thyroid storm in this setting is sporadic. Such a case highlights the importance of being aware of atypical presentations of thyroid storm. The association between thyrotoxicosis and Moyamoya disease has been reported. There is no published case report or review describing Moyamoya syndrome in association with the transformation of hypothyroidism to hyperthyroidism.

A 28-year-old patient with a known history of hypothyroidism, type 2 diabetes mellitus (T2DM) on SGLT2 inhibitors, polycystic ovarian syndrome (PCOS) and mixed anxiety-depressive disorder presented to the accident and emergency department with palpitations, chest discomfort and focal seizures reported by her partner and family. Initial blood tests showed euglycemic diabetic ketoacidosis (eDKA) and a very high thyroid function test suggestive of thyrotoxicosis. A CT of the brain showed a developing right frontal lobe infarct. The patient's condition continued to deteriorate despite ICU input. A repeat brain CT suggested Moyamoya syndrome. The patient died despite early detection and thorough, multidisciplinary care input from endocrinology, neurology, stroke and critical care teams.

This example underpins the poor prognosis of Moyamoya syndrome, even with early identification and management, the need for multispecialty cooperation, and the difficulties in treating the uncommon complication of thyrotoxicosis.

## Introduction

Thyrotoxicosis is a potentially life-threatening complication of hyperthyroidism. Thyrotoxic crisis can show a variety of systemic symptoms and cardiovascular events, such as palpitations, chest pain and fever. Neurological presentation is less common but clinically important; patients may present with a wide range of cerebrovascular and neurological symptoms, such as confusion, altered mental state and seizures [1]. It is less common but should be taken into consideration in the differential diagnosis of any patient with thyrotoxicosis and neurological deterioration secondary to encephalopathy, which can manifest as encephalopathy, focal or generalised seizures [2]. This case highlights a rare and clinically significant transition from hypothyroidism to thyrotoxicosis. Such a transition is uncommon; several reports of such progression are often linked to dose replacement or fluctuation in autoimmune activities. 

The thyroid storm can cause hypercoagulability, which may increase the risk of cerebrovascular events such as haemorrhage or infarction due to endothelial dysfunction and increased metabolic demand resulting from an excess of thyroid hormones [3].

Furthermore, euglycemic diabetic ketoacidosis (eDKA), which is characterised by ketoacidosis without significant hyperglycaemia, is frequently made worse by drugs like SGLT2 inhibitors [4], which are becoming more commonly used in recent practice. 

This rare association becomes even more critical when compounded by cerebrovascular complications such as Moyamoya syndrome, which further worsens prognosis and highlights the need for early recognition and multidisciplinary management.

## Case presentation

A 28-year-old female patient presented to the accident and emergency with chest discomfort associated with palpitations, sweating, left arm paraesthesia, weakness, and exhaustion.

The patient described an approximately one-month history of having focal seizure activities that mostly affected her left arm, followed by a post-ictal state. She had experienced six seizures in one month, four of which were on her left side, and the seizure frequency had been rising. The patient also described a history of exhaustion, palpitations, and unintentional weight loss.

Past medical history included hypothyroidism (treated with levothyroxine 2018-2021), type 2 diabetes mellitus (T2DM; treated with metformin and SGLT2 inhibitors), mixed anxiety-depressive disorder and polycystic ovarian syndrome (PCOS). No history of alcohol excess or illicit drug use. 

Initial medical assessment and clinical examination were unremarkable. Blood pressure was 131/64 mmHg, pulse was 94 beats per minute (bpm), temperature was 37.1°C, respiration rate was 24 breaths per minute, and oxygen saturation was 94% on air.

Laboratory results confirmed thyrotoxicosis, indicated by a significantly suppressed thyroid-stimulating hormone (TSH <0.01 µU/mL; reference range 0.27-4.20 µU/mL) and elevated free thyroxine (T4 >100 pmol/L; reference range 11.3-21.6 pmol/L). Plasma ketones were markedly elevated at 6.2 mmol/L (reference range 0.1-0.6 mmol/L), while blood glucose remained within normal limits at 8.1 mmol/L. Venous pH was 7.29 (reference range 7.35-7.45), consistent with eDKA. The investigation suggested eDKA; it was important to consider other possible causes of the metabolic acidosis, such as recent seizures, underlying infection, reduced oral intake, alcohol use, or a drug overdose, which could also contribute, so each of these possibilities was explored through the patient’s history, examination, and laboratory results before confirming the diagnosis.

**Figure 1:**
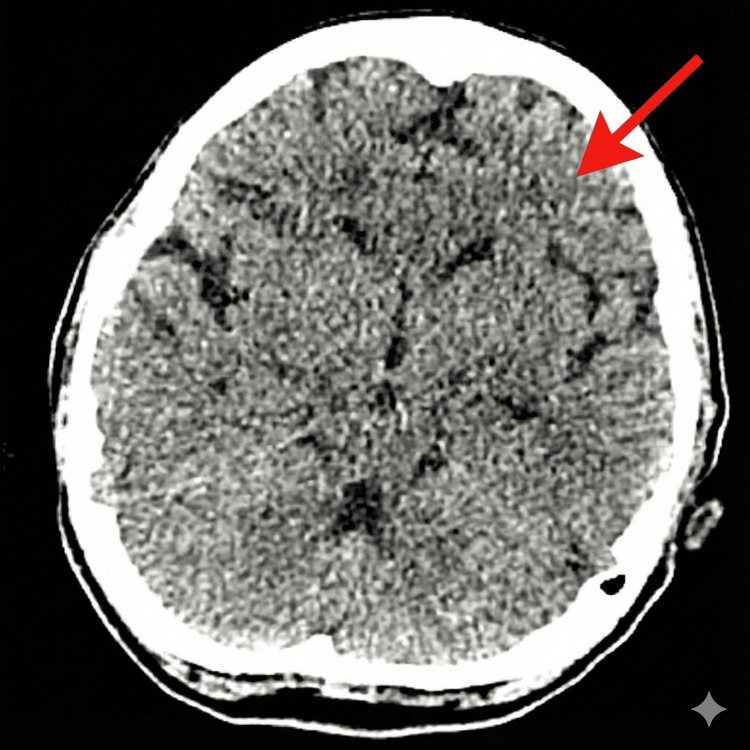
A CT of the brain demonstrating features of hypodensities in the frontal lobe.

The initial management plan was the ABCD (Airway, Breathing, Circulation, Disability) approach to stabilise metabolic and cardiovascular conditions. Intravenous fluids, beta-blockers, and the anti-thyroid drug propylthiouracil were administered on admission. A multi-disciplinary team (MDT) approach to management started on admission, including the ICU and endocrinology team input. The patient was admitted to the ICU for monitoring and further input from endocrinology, neurology, and the stroke team. A brain CT scan showed extensive dense calcification of the falx, which is unusual in a patient of this age (Figure 1).

The following day, the patient's condition deteriorated, which required intubation and artificial ventilation when the Glasgow Coma Scale (GCS) dropped to 6-7/15. A CT angiography showed no signs of major artery occlusion. 

**Figure 2:**
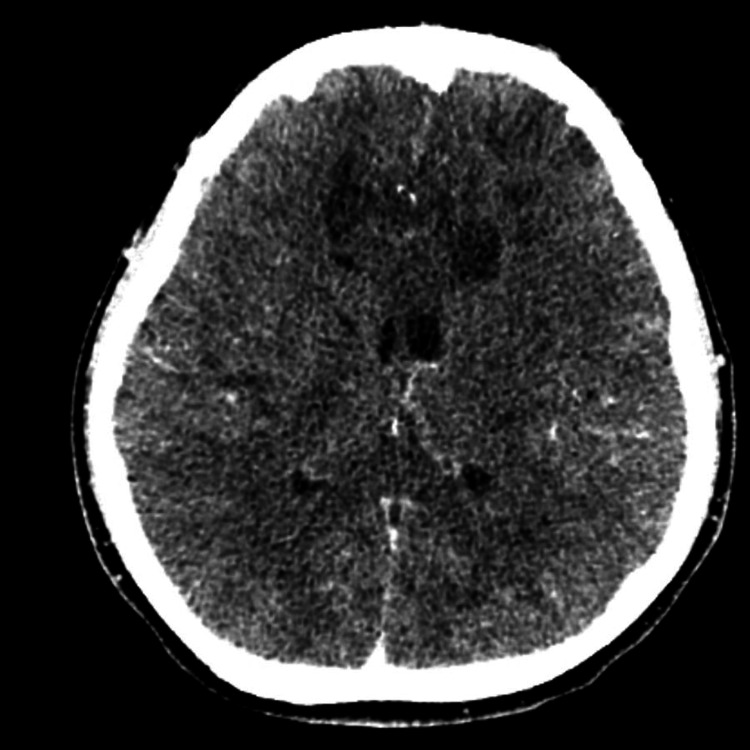
Extensive bilateral frontoparietal hypodensities along with effacement of the cerebral sulci and mild effacement of the lateral ventricles due to oedema.

A repeat CT brain showed extensive bilateral frontoparietal hypodensities along with effacement of the cerebral sulci and mild effacement of the lateral ventricles due to oedema (Figure 2). 

Nevertheless, given the bilateral pattern of the infarcts and the potential for vascular alterations triggered by thyroid crisis, the working diagnosis changed to Moyamoya syndrome after neurology input. 

The patient remained unstable during her ICU admission, necessitating mechanical ventilation and high-dose sedation. She experienced growing neurological impairments, such as dilated and fixed pupils. Neurological brain death was diagnosed after a follow-up CT scan revealed a fresh intraventricular haemorrhage together with indications of brainstem herniation.

## Discussion

This case presents a rare combination of transformation of hypothyroidism to thyrotoxic crisis, eDKA, and acute cerebrovascular events (Moyamoya syndrome), which together contributed to a poor outcome despite aggressive and multi-disciplinary management.

Thyrotoxic crisis, or thyroid storm, is a medical emergency typically triggered by a precipitating factor such as infection, surgery, or trauma in patients with untreated or inadequately treated hyperthyroidism. Clinical features include fever, tachycardia, agitation, confusion, and multi-organ dysfunction, including cardiac arrhythmias, liver dysfunction, and metabolic acidosis [5]. The pathophysiology of thyroid storm includes heightened metabolic demands, catecholamine excess, and, in some cases, acute cerebrovascular complications, including encephalopathy and seizure activity.

eDKA, the triad of the presence of ketoacidosis without significantly elevated blood glucose levels [6], and the increasing use of SGLT2 inhibitors [7] have been recognised as rare complications. This condition is associated with normal or slightly elevated blood glucose levels, which can mask the underlying ketoacidosis. The mechanism is likely related to the inhibition of renal glucose reabsorption, which decreases blood glucose levels but does not prevent the development of ketosis [8].

Cerebrovascular complications can include ischaemic and haemorrhagic strokes due to a hypercoagulation state and endothelial damage. This case raises suspicion of Moyamoya syndrome, a rare cerebrovascular condition characterised by progressive stenosis or occlusion of the intracranial arteries and development of collateral vessels [9, 10]. Thyrotoxicosis may predispose to stroke [11] through several mechanisms, including increased blood viscosity, endothelial dysfunction, and hypercoagulability. Patients with an underlying cause, such as thyrotoxicosis disorder, are classified as having Moyamoya syndrome, whereas those without an identified cause are classified as having Moyamoya disease [12]. 

The MDT approach is crucial for managing such infrequent team presentations. The stroke, neurology, endocrinology, and intensive care teams were all involved in the case's management. Close observation of the patient's condition and early escalation of treatment were crucial in giving the assistance required to manage this complicated and quickly changing situation. Specialists in neurology and stroke were able to identify possible cerebrovascular reasons early on, which helped guide the right imaging and therapies.

The medical staff communicated openly with the patient's family about the poor prognosis and end-of-life choices when the patient's neurological state worsened, and brain death had been verified. This emphasises how crucial it is to give compassionate care and make sure families participate in decision-making at important times.

## Conclusions

The report presented a very rare case of Moyamoya syndrome with thyroid storm in a patient with a background of hypothyroidism. Thyroid storm is a rare but life-threatening endocrine emergency; its development following conversion from hypothyroidism to hyperthyroidism is exceptionally rare. Notably, out of the available literature, none of the reported cases involve a patient who moved from hypothyroidism into hyperthyroidism before developing thyroid storm. Most cases begin with Graves’ disease or thyroiditis, where hyperthyroidism is the starting point. This makes the sequence seen in this case of hypothyroidism, followed by thyrotoxicosis and then thyroid storm in the context of developing Moyamoya disease, particularly unusual, and perhaps this is the first case in the published literature. 

Moyamoya syndrome is a devastating neurological outcome resulting from progressive narrowing and occlusion of cranial arteries, including carotid arteries. Thyrotoxic crisis can worsen vasoconstriction and cerebrovascular events through several mechanisms. Doctors must maintain a high index of suspicion for cerebrovascular involvement in thyrotoxic patients. If neurological symptoms are present, a full neurological examination should be considered, and immediate screening via CT angiogram or MRI angiogram should be considered, as identifying Moyamoya vasculopathy fundamentally changes management. The significance of early diagnosis and management of thyrotoxic crisis, knowledge of the uncommon side effect of eDKA in patients taking SGLT2 inhibitors, the function of neurovascular consultations in the context of cerebrovascular complications, and the necessity of a coordinated, multispecialty approach in critical care are the main lessons to be learnt. Despite receiving optimal care, this example shows that some patients may not survive even after rigorous, multispecialty management because of the severity and complexity of their underlying diseases. A more standardised approach to treating such complex presentations and additional study into the biology of uncommon disorders linked to thyrotoxicosis may assist in increasing survival rates and the standard of care for people with comparable conditions.
